# The complete mitochondrial genome of a medical important wasp, *Vespa magnifica* (Hymenoptera, Vespidae)

**DOI:** 10.1080/23802359.2021.1981163

**Published:** 2021-12-23

**Authors:** Xi Feng, Binqiang Xu, Yan Huang

**Affiliations:** a Changsha Hospital of Hunan Normal University, Changsha, China; b The Fourth Hospital of Changsha, Changsha, China; c Institute of Emergency and Critical Care Medicine of Changsha, Changsha, China

**Keywords:** Mitogenome, Vespidae, venom, sting, phylogenetic

## Abstract

*Vespa magnifica* (Smith) is an aggressive social wasp species of Vespidae family. This species is of medical importance for its dangerous sting, traditional medicinal use and valuable venom components. Here, a complete mitogenome of *V. magnifica* was presented. It was 16,730 bp in length with nucleotide composition of AT: 79.4% and CG: 20.6%. In total, 13 protein-coding genes (PCGs), 22 transfer RNA, and two ribosomal RNA genes were annotated in this mitogenome. Phylogenetic analysis was performed using *V. magnifica* with 20 other species of Vespidae. The result indicated those species of genus *Vespa* fell into a paraphyletic group. Moreover, the *Vespa* species with large body size were clustered into a clade. This mitogenome resource can contribute to further phylogenetic and taxonomic study on genus *Vespa*.

Stings of wasps are common outdoor risks to people who disturb those aggressive insects deliberately or accidentally. Clinical syndromes following the sting are often characterized by itching, swelling, and acute pain. Allergic reactions with serious clinical outcomes are also commonly reported from emergency department globally. *Vespa magnifica* (Vespidae) is an aggressive wasp species with large body size. Cases of serious syndrome after sting of this species were reported from Asia countries (Vikrant et al. 2005; George et al. 2008; Vikrant and Parashar 2017). Meanwhile, the raw venom of *V. magnifica* is a traditional medicine for rheumatoid arthritis, which was employed by Jingpo ethnic group of Yunnan Province, China (Zhou et al. 2019). Many researches had revealed valuable components from the venom of *V. magnifica*, such as bioactive peptide, anticoagulant serine protease, kininogen, etc (Xu et al. 2006; Han et al. 2008; An et al. 2012). In Yunnan Province of China, *V. magnifica* together with other species *Vespa mandarinia* and *Vespa ducalis*, were all named as ‘da tu feng,’ which particularly emphasizes their large body size, dangerous sting, as well as similar morphological characters. Considering the potentially medical importance of *V. magnifica*, a complete mitochondrial genome sequence of *V. magnifica* (GenBank: MT137097.2) was provided here.

The female *V. magnifica* samples were captured by Zichao Liu in Xinzhai Village, Lvchun County, Yunnan Province, China (102°50′N, 22°80′E). The wasps were attracted by baits of chicken meat and captured by insect nets. Then they were deposited into pure ethyl alcohol for storage. The species identification was performed following the description of its morphological characters (Rao et al. 2014). The voucher specimen of *V. magnifica* used in this study was assigned with a unique series code (MG20201101-3) and deposited into the bio-sample herbarium of Institute of Emergency and Critical Care Medicine of Changsha. The thorax part of wasp was detached using cleaned fine forceps and muscle of voucher sample was transferred to a DNAase-free tube. Then raw DNA material was extracted using TIANamp Genomic DNA Kit (DP304) (TIANGEN BIOTECH, Beijing, China). The DNA material was processed and qualified following the instruction of Illumina sequencing platform. The PE150 libraries were prepared using TruSeq Nano DNA LT Sample Preparation Kit (Illumina, San Diego, CA, USA).

Sequencing was performed on the Illumina HiSeq X Ten platform (Illumina Inc., San Diego, CA, USA) using the prepared DNA libraries. After sequence filtering by SOAPnuke (version: 1.3.0) (Chen et al. 2018), 2.5 Gb clean reads were finally assembled into a complete circular mitochondrial genome of 16,730 bp in length using MITObim (v1.8) (Hahn et al. 2013). The sequence was deposited into GenBank and given a unique accession number MT137097.2. The nucleotide composition was calculated using MEGA 7 software, which showed AT: 79.4% and CG: 20.6% (T:40.7%, A:38.7%, C:14.7%, G:5.9%) (Kumar et al. 2016). The protein-coding gene (PCGs) annotation and the prediction of tRNAs secondary structure (except for tRNA-Asn and tRNA-Ser) were performed by applying Mitochondrial Genome annotation 2 (MITOS2) webserver (Bernt et al. 2013). In total, 13 PCGs, 22 tRNA, and two rRNA genes were annotated in this mitogenome. The open reading frames (ORF) features of all the 13 PCGs were recognized using online version of ORFfinder (https://www.ncbi.nlm.nih.gov/orffinder/).

For phylogenetic analysis, a Bayesian tree was constructed based on PCGs and two rRNA genes of *V. magnifica* and 20 other species of Vespidae. The MrBayes (v3.2.4) was used for this analysis. The chain length was set as 5,000,000 generations and sampled every 1000 generations during the calculation (Ronquist et al. 2012). *Eustenogaster scitula* was set as outgroup (Figure 1). The result of phylogenetic analysis was largely consistent with previous reports (Kim et al. 2017; Liu et al. 2020). All the species of genus *Vespa* fell into a large group which was paraphyletic. In addition, the species with large body sizes, including *V*. *magnifica*, *V*. *mandarinia,* and *V*. *ducalis*, were clustered into a clade. The present mitogenome of *V. magnifica* could contribute to the phylogenetic studies of the genus *Vespa* and the identification of large wasp species identification.

**Figure 1.**
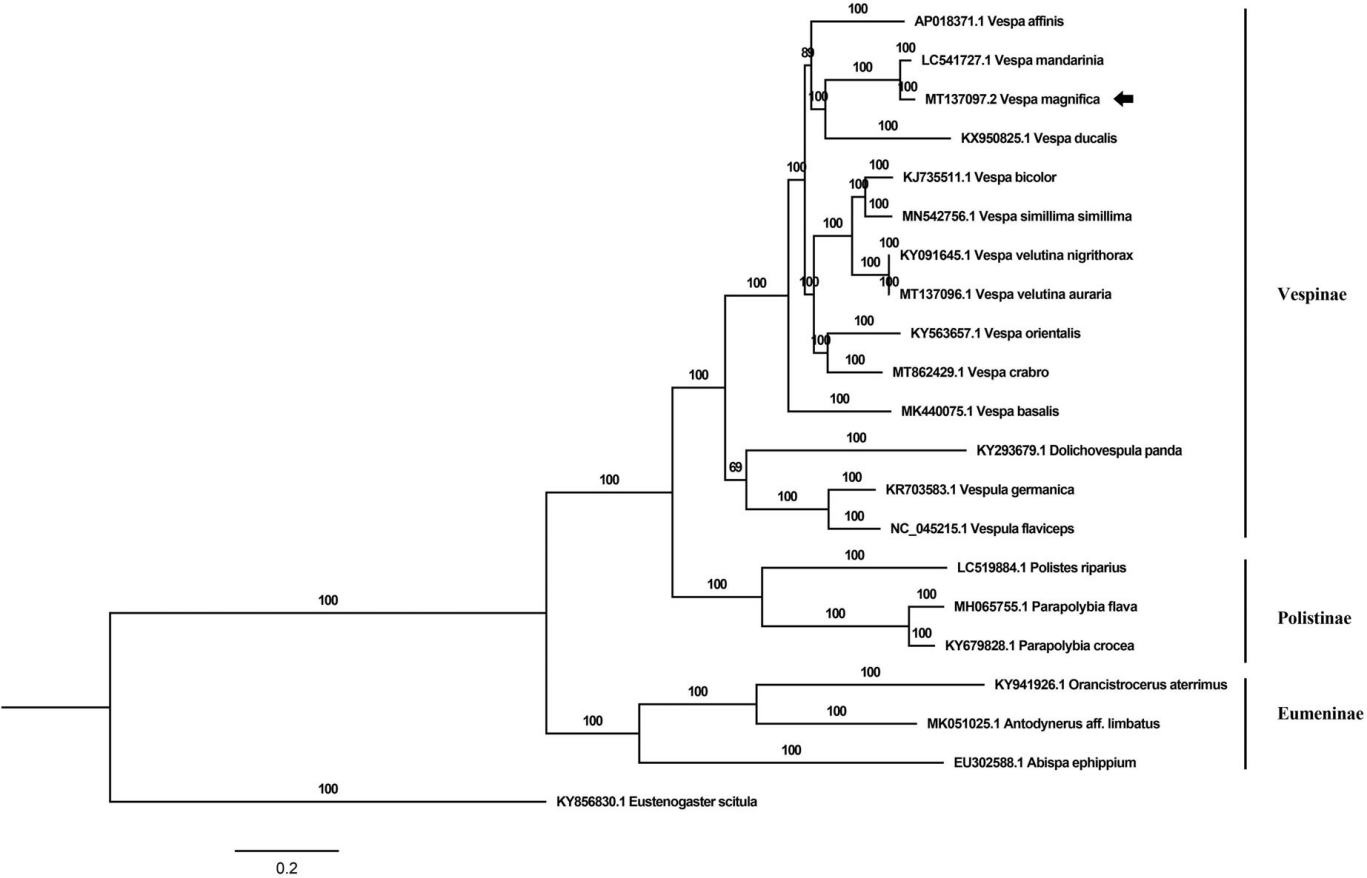
The phylogenetic tree was constructed based on PCGs genes and rRNA genes sequences of *V. magnifica* with other 20 species of Vespidae by using MrBayes, four chains for 5,000,000 generations, sampled each 1000 generations.

## Data Availability

The genome sequence data that support the findings of this study are openly available in GenBank of NCBI at [https://www.ncbi.nlm.nih.gov] under the accession no. MT137097.2. The associated BioProject, SRA, and Bio-Sample numbers are PRJNA703057, SRS8300133 and SAMN18011289 respectively.
